# Projection of diabetes morbidity and mortality till 2045 in Indonesia based on risk factors and NCD prevention and control programs

**DOI:** 10.1038/s41598-024-54563-2

**Published:** 2024-03-05

**Authors:** Mugi Wahidin, Anhari Achadi, Besral Besral, Soewarta Kosen, Mardiati Nadjib, Atik Nurwahyuni, Sudarto Ronoatmodjo, Ekowati Rahajeng, Masdalina Pane, Dian Kusuma

**Affiliations:** 1 Doctoral Program of Public Health, Faculty of Public Health, Universitas Indonesia, Depok, Indonesia; 2 National Research and Innovation Agency, Jakarta, Indonesia; 3 Universitas Esa Unggul, Jakarta, Indonesia; 4 Faculty Public Health, Universitas Indonesia, Depok, Indonesia; 5 National Institute of Health Research and Development, Ministry of Health, Jakarta, Indonesia; 6 Department of Health Services Research and Management, School of Health and Psychological Sciences, University of London, London, UK

**Keywords:** Diabetes, Projection, Prevalence, Mortality, Diseases, Endocrinology, Health care, Medical research, Risk factors

## Abstract

Diabetes Mellitus is one of the biggest health problems in Indonesia but the research on the disease’s projection is still limited. This study aimed to make a projection model of prevalence and mortality of diabetes in Indonesia based on risk factors and NCD programs. The study was a quantitative non-experimental study through multiple linear regression models and system dynamics. The baseline projection was created by 2018 data and projections until 2045 involved the dynamization of risk factors and programs, population, and case fatality rate. The model was created from 205 districts data. This study used secondary data from Basic Health Research, BPJS Kesehatan, NCD programs, and Ministry of Health. The prevalence of diabetes in Indonesia is estimated to increase from 9.19% in 2020 (18.69 million cases) to 16.09% in 2045 (40.7 million cases). The prevalence will be lower to 15.68% (39.6 million) if interventions of programs were carried out, and to 9.22% (23.2 million) if the programs were added with prevention of risk factors. The projected number of deaths due to diabetes increases from 433,752 in 2020 to 944,468 in 2045. Deaths due to stroke among diabetes increases from 52,397 to 114,092 in the same period. Deaths from IHD among diabetes increase from 35,351 to 76,974, and deaths from chronic kidney disease among diabetes increase from 29,061 to 63,279. Diabetes prevalence and mortality in Indonesia rise significantly in Indonesia and can be reduced by intervention of several programs and risk factors. This study findings could be source of planning and evaluation of Diabetes prevention and control program at national and provincial level in the future related to risk factors control and program development.

## Introduction

The burden of Diabetes, one of main Non-Communicable Diseases (NCD), in term of prevalence and mortality becomes a serious problem in the word as well as in Indonesia. The prevalence rate of the disease was 5943 per 100.000 population in 2019 worldwide, which rose from 2968 per 100,000 in 1990. Meanwhile, the mortality rate was 20.5 per 100,000 population which increased from 12.37 per 100,000 in 1990^1^. In Indonesia, the prevalence was 5.7% among adults in 2007 became 6.9% in 2013 and 8.5% in 2018^2–4^.

There was common risk factors related to Diabetes such as obesity, smoking, unhealthy diet, lack of physical activity, hypertension, raised in blood glucose, and increase of cholesterol^5–7^. Similarly, study of Peters et al.^8^ revealed that unhealthy diet, lack of physical activity, smoking, hypertension, and obesity. The study of Kristianita described that there was a significant relationship between fruit and vegetable consumption, physical activity, and the incidence of type 2 Diabetes. Moreover, the study of Zhang et al.^9^ stated that overweight, obesity, high triglyceride, and hypertension are risk factors for Diabetes in men and women.

Diabetes becomes part of main disease prevention and control program in Indonesia that is included in the National Medium-Term Development Plan 2020–2024^10^, Strategic Plan of the Ministry of Health 2020–2024^11^, and indicators in the Minimum Service Standards for district government^12^. Thus, there are several NCD programs developed nationally namely NCD integrated post (Posbindu), NCD integrated Service in Primary Health Center (Pandu), as well as the Chronic Disease Service (Prolanis) program^7^. Furthermore, there are also screening program including obesity, central obesity, and program of Diabetes standard services in the minimum service standards (SPM) as obligation of district government^12^. The program might influence the development of diabetes, but information of the influence was still limited.

In order to develop adequate prevention and control programs of diabetes, projection of the diseases, especially morbidity and mortality is needed. Projection of Diabetes prevalence may be developed using risk factors and prevention and control programs. The research of Meng et al.^13^ included risk factors used for diabetes projection, namely body mass index, smoking, alcohol consumption, physical activity, and meat, fish, and vegetable consumption. Meanwhile, the research of Nai-Arun and Moungmai, used smoking, alcohol consumption, family history of diabetes, family history of hypertension, weight, BMI, blood pressure, age, and sex as predictors of^14^.

Projection of the prevalence and mortality of diabetes mellitus is still limited in Indonesia. One of the Diabetes burden study in Indonesia was conducted in 1993, which showed that the Diabetes treatment burden in Indonesia reached IDR 1.5 billion per day or IDR 500 billion a year^15^. Another study was a projection till 2024 by Nurhayati^16^ that by 2020 the prevalence of Diabetes in Indonesia was 8.71% which rose from 8.13% in 2017 and becomes 9.49% in 2024. This study was a literature review based on Institute of Health Metric and Evaluation (IHME) data. Compared to the existing data, the prevalence of 2017 was lower than 2018 national data (8.5%)^4^.

Diabetes burden projection research has been conducted in various countries. Research by Tan et al.^17^ in Singapore on the projection of Diabetes complications in 2050 in the form of acute myocardial infarction is estimated to increase from 9300 in 2019 to 16,400 in 2050. The number of strokes increased from 7300 to 12,800, and the number of end-stage kidney disease from 1700 to 2700^17^. Research by Rowley, et al. (2017) on Diabetes projections in the United States through 2030 shows that Diabetes prevalence increases by 54% or more than 54.9 million population between 2015 and 2030, with Diabetes—related deaths increasing 38% to 385,800 people per year^18^. Another Diabetes projection study by Boyle et al.^19^ in the United States shows that the incidence of DM is expected to increase from 8 cases per 1000 in 2008 to 15 by 2050.

Besides influence of the risk factors, influence of prevention and control program to diabetes is not known clearly. The projection is needed to estimate the burden and develop anticipated prevention and control program. The previous projection of diabetes in Indonesia used regression based on risk relative of the risk factors only. Thus, we conducted the study to develop projection of diabetes prevalence and mortality based on risk factors and NCD prevention and control programs in Indonesia. The projection of diabetes using modeling of risk factors and NCD control programs in this manuscript is the first method in Indonesia.

## Methods

### Study design

The study used a quantitative non-experimental study design through developing projection models with multiple linear regression and *system dynamics*. The model was based on risk factors and Diabetes prevention and control programs, as well as population size, Diabetes risk factor growth, Diabetes prevention and control program growth, case fatality rate, and population projection. This study used secondary data from Basic Health Research 2007, 2013, 2018, National Health Insurance Body (BPJS) 2016–2020, Directorate of P2PTM Ministry of Health 2016–2020, Center for Data and Information the Ministry of Health (2019–2021), and the Central Bureau of Statistics. Wes use district level as analysis unit. This study used risk factors and program of Diabetes to project the morbidity and mortality of Diabetes which was a new method of analysis in projection of the disease in Indonesia. The previous study in Indonesia used only risk factors and existing cases to project the disease.

### Study approval

The study was approved by Universitas Indonesia and the authors confirmed that all methods were performed in accordance with the relevant guidelines and regulations in Universitas Indonesia.

### Dependent variable

The dependent variables were diabetes morbidity and mortality. Morbidity means prevalence of diabetes, which was defined as percentage of adult respondent (15 years and above) who had diabetes based on medical doctor diagnosis which was adjusted by the prevalence of diabetes based on blood glucose measurement (8.5%). The mortality was number of deaths due to diabetes, number of death due to stroke among diabetes cases, deaths due to ischemic health disease among diabetes cases, and deaths due to chronic kidney disease among diabetes cases.

### Independent variables

There were 10 risk factors and 8 NCD prevention and control programs included in the projection model. The risk factors consisted of prevalence of overweight, obesity, central obesity, sweet food consumption, sugary beverage consumption, fatty food consumption, lack of fruit and vegetable consumption, lack of physical activity, smoking, and hypertension. Meanwhile, the prevention and control programs included Posbindu, village running Posbindu, examination of Posbindu, Pandu, Prolanis, routine checking blood glucose, Minimum standard service (SPM) for Diabetes services and minimum standard services of NCD screening.

Overweight was categorized by body mass index (BMI) for 25–26.9, meanwhile 27 and above for obesity. Central obesity category was waist circumferences 90 cm (males) and 80 and above (females). Sweety food consumption was consumption the food containing excessive sugar/carbohydrate 1 time or more a day and sweety beverage consumption was consumption the drinking water containing excessive sugar 1 time or more a day. Fatty food consumption was consumption excessive fat/fried food 1 or more a day. Lack of fruit and consumption was no consumption of or less than 5 portions of fruit or vegetables a day. Moreover, Lack of physical activity was less 30 min or 150 min moderate physical activity a day. Smoking meant active smoking in the last month. Meanwhile, hypertension was based on blood pressure examination for those who has systole of 140 mmHg or diastole for 90 mmHg.

Posbindu was a community participation on detecting and monitoring NCD risk factors. Village running Posbindu meant village that has active Posbindu. Examination of Posbindu was activity of NCD risk factors detection namely smoking, physical activity, fruit and vegetable consumption, weight and height measurement, blood pressure measurement, and blood measurement. Pandu was an activity of detection of NCD risk factors, detection of NCD cases and standard treatment in primary health centers. Prolanis is a chronic disease management, including diabetes and hypertension, run by primary health center with activities of monthly blood glucose measurement, blood pressure measurement, treatment, physical activity, and counseling.

Coverage of village running Posbindu was percentage of village had Posbindu, coverage of Posbindu examination was percentage of members examined in the Posbindu. Coverage of Pandu is percentage of Primary health center (PHC) developing integrated NCD, coverage of Prolanis was member of Prolanis among people aged 15 years and above. Routine of blood glucose checking was percentage of people who regularly checks blood glucose monthly. Coverage of SPM Diabetes service was percentage of Diabetes patients have standard treatment, and coverage of NCD screening was people aged 15 years and above who have complete screening for NCD risk factors.

### Data analysis

Data analysis performed in the study was development of baseline prevalence and mortality projection in 2018 using multiple linear regression and projections till 2045 using system dynamics. Multiple Linear regression was developed through step of bivariate selection, multivariate modelling, and final model development^20,21^. Bivariate selection was performed by correlation analysis between risk factors and diagnosed Diabetes prevalence, which risk factors that had p value less than 0.25 was inputted into full model^20^. Based on bivariate analysis, 16 out of 18 predictors were included in the full model. Two risk factors namely sweety food consumption and sweety beverage consumption were excluded.

The multivariate testing used Enter method. Then, the multivariate modelling was performed by excluding variables from full model that had p value more than 0.05. If the variable did not influence R^2^ and B of other variables for 10%, the variables were kept excluded. The variables excluded from final model were Pandu and lack of fruit and vegetables consumption. So, there were 14 determinants included in the final model. In order to justify the fit of the model, all assumption of multiple linear regression were tested, for existence, independence, linearity, homoscedasticity, normality, and collinearity^20,21^ After testing, all assumption were complied.

Based on multiple linear regression analysis, there were 9 variables consisting of 4 risk factors and 5 prevention and control programs as predictors of Diabetes prevalence in the final model. With R^2^ 0.571, the model described as Diabetes prevalence = − 1.212 + 0.216 overweight prevalence + 0.017 obesity prevalence + 0.112 central obesity prevalence + 0.019 prevalence of fatty food consumption–0.001 Percentage of villages with PTM Posbindu + 0.003 percentage of Pandu + 1.510 prevalence of routine blood sugar checks−0.012 SPM coverage of DM Diabetes service + 0.008 SPM coverage productive age screening.

In order to make a projection to 2045, we incorporate trend/dynamization of each risk factor and program. Risk factors’ trend based on their trends from 2007 to 2018 based on Basic Health research Data. Trend of the program based on data from 2016 to 2020. Assumption of SPM of Diabetes health services and SPM of productive age screening using random normal based on the average of 3 years (2019–2021) and its standard deviation.

Assumptions of case fatality rate of diabetes and proportion of its complication were based on BPJS data 2016–2020. Case fatality rate of Diabetes was 2.32% for diabetes, proportion of death due to stroke, ischemic heart disease, and chronic kidney disease among Diabetes cases was 12.08%, 8.15%, and 6.7% respectively. Meanwhile, assumption of neuropathy due to diabetes was 53.64%^22^, retinopathy was 30.7%^23^, and Diabetes Keto Acidosis (DKA) among diabetes cases was 3,07%^24^ and its mortality for 72 h was 28.57%^25^. Projection was made in three scenarios, namely scenario without intervention (scenario 1), scenario with program intervention of village with Posbindu and SPM of Diabetes services each 100% coverage (scenario 2), and scenario with program intervention and halt of the rate of risk factors (overweight, obesity, central obesity, and fatty food consumption) as condition in 2018. The projection results have been declared valid after discussion with experts and have an Absolute Mean Percentage Error (MAPE) of 12% for provincial and national projections and 23% for district/city projections^26^. For generating maps, Looker Studio software with release date on 20 December 2022 was used in this geographical distribution analysis using results of this study. The software could be accessed at https://lookerstudio.google.com/overview.

### Ethics approval and consent to participate

The study was approved by The Research and Community Engagement Ethical Committee Faculty of Public Health Universitas Indonesia No. Ket-438/UN2.F10.D11/PPM.00.02/2022 on June 22^nd^ 2022. The data (aggregate data) used in this study were anonymized before its use. Following The Guideline and Ethical Standard of National Health Research and Development issued by Ethical Board of National Health Research and Development, Ministry of Health (2013), this study did not use informed consent as it used secondary data. The authors had permission to use the data from each secondary data holder, namely Head of Policy and Development Body, Ministry of Health, Director of Non-Communicable Disease Prevention and Control, Ministry of Health, and Director of National Health Insurance (BPJS).

### Consent for publication

We, the authors, give our consent for the publication of this paper, which can include detail of tables and figures to be published in Scientific Reports. Data of diabetes prevalence and ant the risk factors 2007 can be accessed at https://labmandat.litbang.kemkes.go.id/adownload/?id=2&lkey=82206a9b1521b38 (closed), 2013 at https://labmandat.litbang.kemkes.go.id/adownload/?id=3&lkey=4c9c023be7c4a12 (closed), and 2018 at https://labmandat.litbang.kemkes.go.id/adownload/?id=4&lkey=8ad2f351fd26042 (closed). Data of Indonesia population can be accessed at https://www.bps.go.id/subject/12/kependudukan.html#subjekViewTab5 (open). Links of diabetes risk factors were granted from Head of Policy and Development Body, Ministry of Health. Data of national NCD programs supplied by Director of Non-Communicable Disease Prevention and Control, Ministry of Health. Data of diabetes mortality was supplied by Director of National Health Insurance (BPJS).

## Results

### Projection of diabetes prevalence

**Figure 1:**
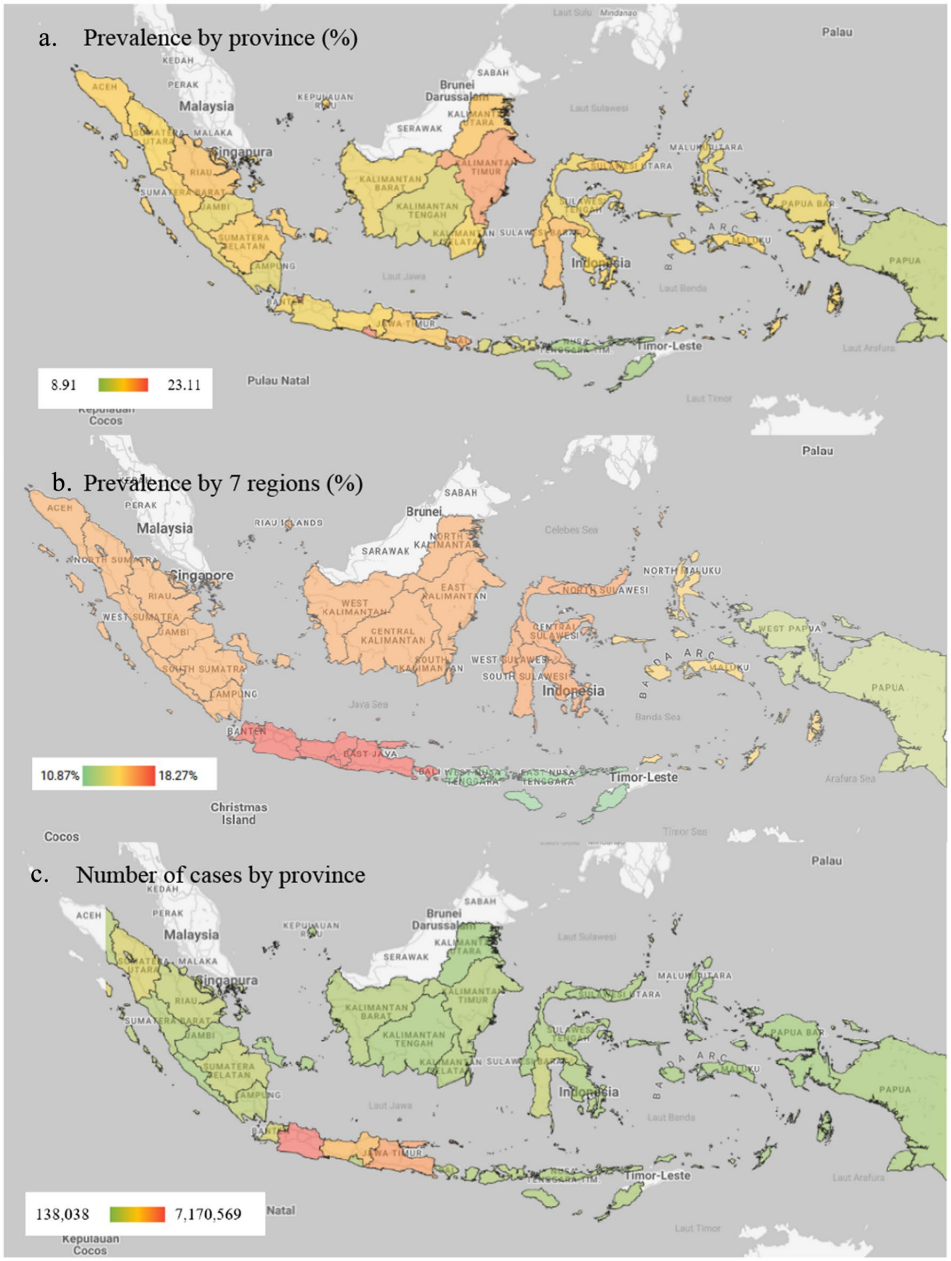
Projection of Morbidity of Diabetes in Indonesia 2045.

The prevalence and number of Diabetes cases (total) in Indonesia and in each province is estimated to increase quite high in 2020–2045. Nationally, Diabetes prevalence increased from 9.19% in 2020 (18.69 million cases) to 16.09% in 2045 (40.7 million cases). It rose 75.1% over 25 years, with an average increase of 3% from prevalence per year. The provinces with the highest prevalence in 2045 are Jakarta (23.11%) and the lowest East Nusatenggara province (8.91%) (Fig. 1a, Table 1). Based on seven regions, Java-Bali region had the highest average of Diabetes prevalence (18.27%) and the lowest was Nusatenggara region (10.87%) (Fig. 1b). The most cases in 2045 are in West Java Province (7,170,569 cases) and the lowest in North Kalimantan Province (138,038 cases) (Fig. 1c, Table 2). The microvascular complication of diabetes, namely neuropathy and retinopathy were also projected to rise from 2020 to 2045. Neuropathy increased from 10,028,638 cases to 21,836,747 cases and retinopathy rose from 5,739,732 cases to 12,497,915. The highest cases of 2045 were in West Java province with 3,846,293 cases and 2,201,365 and the lowest was in North Kalimantan Province with 74,044 cases and 42,378 cases for neuropathy and retinopathy, respectively (Tables 3 and 4).

**Table 1:** Projection of DIABETES PREVALENCE IN Indonesia, 2020–2045, by province.

No	Province	Diabetes prevalence projection (%)					
		2020	2025	2030	2035	2040	2045
1	Aceh	8.31	9.87	11.45	13.14	14.74	16.39
2	North Sumatera	8.84	10.34	11.72	13.17	14.76	15.83
3	West Sumatera	8.42	9.99	11.53	13.35	14.94	16.55
4	Riau	8.58	10.40	12.26	14.08	15.81	17.70
5	Jambi	6.77	8.09	9.51	10.83	12.43	14.28
6	South Sumatera	7.35	9.15	11.01	12.90	14.92	16.83
7	Bengkulu	7.17	8.64	9.68	10.98	12.47	14.49
8	Lampung	6.63	8.10	9.61	11.05	12.64	13.86
9	Bangka Belitung Island	9.66	11.07	12.69	14.23	15.52	17.16
10	Riau Island	9.54	10.91	12.29	13.73	15.15	16.47
11	Jakarta	14.30	16.09	18.12	19.60	21.40	23.11
12	West Java	9.42	10.56	11.62	12.78	13.90	14.95
13	Central Java	8.99	10.44	11.73	13.23	14.50	15.86
14	Yogyakarta	12.60	14.31	16.08	17.84	19.99	21.94
15	East Java	10.07	11.55	12.86	14.08	15.51	17.01
16	Banten	9.66	10.58	12.22	13.18	14.43	15.56
17	Bali	10.31	12.25	13.93	15.87	17.31	19.43
18	West Nusa Tenggara	6.58	7.41	8.97	10.06	11.64	12.84
19	East Nusa Tenggara	3.89	5.10	6.10	6.90	8.09	8.91
20	West Kalimantan	7.28	8.76	9.76	11.39	13.16	14.48
21	Central Kalimantan	7.43	8.50	9.65	10.89	12.01	12.97
22	South Kalimantan	8.89	10.27	11.52	12.88	14.23	15.42
23	East Kalimantan	12.36	13.70	15.23	17.01	18.58	20.25
24	North Kalimantan	14.33	14.83	15.64	16.18	16.53	17.29
25	North Sulawesi	10.61	11.76	12.82	13.46	14.85	15.84
26	Central Sulawesi	9.12	10.30	10.99	12.18	13.31	14.72
27	South Sulawesi	8.62	10.64	12.25	14.37	16.07	17.99
28	Southeast Sulawesi	8.22	9.59	10.99	12.65	14.20	15.68
29	Gorontalo	10.62	12.26	12.85	14.57	15.88	17.03
30	West Sulawesi	7.47	9.41	11.24	13.27	15.25	17.13
31	Maluku	7.89	9.33	10.90	12.41	13.97	15.55
32	North Maluku	8.84	9.33	10.97	12.12	12.87	14.28
33	West Papua	8.89	9.83	10.99	12.30	12.98	14.28
34	Papua	7.71	8.37	9.08	9.97	10.87	11.19
	Indonesia	9.19	10.61	11.89	13.37	14.79	16.09

**Table 2:** Projection of diabetes cases in Indonesia, 2020–2045, by province.

No	Province	Number of diabetes cases projection					
		2020	2025	2030	2035	2040	2045
1	Aceh	321,498	416,124	519,639	635,349	751,360	873,750
2	North Sumatera	943,618	1,187,416	1,432,263	1,688,543	1,965,634	2,171,341
3	West Sumatera	337,347	430,949	532,139	652,695	765,778	881,138
4	Riau	422,944	577,337	749,983	930,448	1,115,769	1,323,481
5	Jambi	182,506	232,864	288,936	343,965	408,111	480,378
6	South Sumatera	462,350	617,245	789,789	977,323	1,181,214	1,379,270
7	Bengkulu	107,272	138,246	163,836	195,098	229,731	274,375
8	Lampung	423,440	549,083	684,452	817,592	960,440	1,072,210
9	Bangka Belitung	106,364	131,390	160,724	190,355	217,009	248,833
10	Riau Island	162,291	218,131	283,507	362,349	453,336	555,171
11	Jakarta	1,170,540	1,363,348	1,578,110	1,746,904	1,932,776	2,093,705
12	West Java	3,526,676	4,247,308	4,953,851	5,708,318	6,456,651	7,170,569
13	Central Java	2,425,092	2,935,055	3,397,962	3,902,698	4,320,404	4,745,282
14	Yogyakarta	393,501	476,903	568,148	667,693	791,184	916,821
15	East Java	3,184,025	3,768,696	4,285,593	4,769,819	5,287,282	5,784,386
16	Banten	928,816	1,107,764	1,375,654	1,578,329	1,816,134	2,039,772
17	Bali	355,352	454,496	547,063	655,054	745,547	868,706
18	West Nusa Tenggara	250,425	306,043	399,144	478,727	584,753	673,995
19	East Nusa Tenggara	150,977	213,758	274,306	331,772	411,342	474,199
20	West Kalimantan	275,238	356,041	421,828	517,757	623,131	707,526
21	Central Kalimantan	149,556	185,747	225,840	269,995	311,710	348,806
22	South Kalimantan	279,663	348,269	418,653	494,369	569,880	639,636
23	East Kalimantan	342,169	408,152	482,296	565,851	642,241	721,296
24	North Kalimantan	74,716	86,172	100,094	112,312	123,511	138,038
25	North Sulawesi	204,505	237,340	268,411	289,644	324,898	349,627
26	Central Sulawesi	206,043	250,766	286,394	336,462	386,178	445,155
27	South Sulawesi	580,477	753,698	906,079	1,098,762	1,257,503	1,430,337
28	Southeast Sulawesi	158,586	202,326	250,555	309,283	369,209	429,852
29	Gorontalo	94,747	115,651	126,838	148,940	166,522	181,961
30	West Sulawesi	73,876	101,234	130,401	164,058	198,693	232,765
31	Maluku	101,568	128,630	159,333	190,583	223,357	257,124
32	North Maluku	79,931	91,795	116,083	136,318	152,464	177,012
33	West Papua	62,882	79,741	100,719	125,626	146,392	176,556
34	Papua	190,147	224,240	260,512	302,616	344,685	367,506
	Indonesia	18,696,194	22,990,010	27,138,625	31,855,207	36,449,447	40,709,820

**Table 3:** Projection of neuropathy due to diabetes in Indonesia, 2020–2045, by province.

No	Province	Number of diabetes cases projection					
		2020	2025	2030	2035	2040	2045
1	Aceh	172,452	223,209	278,735	340,801	403,029	468,679
2	North Sumatera	506,157	636,930	768,266	905,735	1,054,366	1,164,707
3	West Sumatera	180,953	231,161	285,439	350,106	410,763	472,642
4	Riau	226,867	309,684	402,291	499,092	598,498	709,915
5	Jambi	97,896	124,908	154,985	184,503	218,911	257,675
6	South Sumatera	248,005	331,090	423,643	524,236	633,603	739,840
7	Bengkulu	57,541	74,155	87,882	104,651	123,227	147,175
8	Lampung	227,133	294,528	367,140	438,556	515,180	575,134
9	Bangka Belitung	57,054	70,478	86,212	102,106	116,404	133,474
10	Riau Island	87,053	117,005	152,073	194,364	243,170	297,794
11	Jakarta	627,877	731,300	846,498	937,039	1,036,741	1,123,063
12	West Java	1,891,709	2,278,256	2,657,246	3,061,942	3,463,348	3,846,293
13	Central Java	1,300,819	1,574,364	1,822,667	2,093,407	2,317,465	2,545,369
14	Yogyakarta	211,074	255,811	304,754	358,151	424,391	491,783
15	East Java	1,707,911	2,021,528	2,298,792	2,558,531	2,836,098	3,102,745
16	Banten	498,217	594,205	737,901	846,616	974,174	1,094,134
17	Bali	190,611	243,791	293,444	351,371	399,912	465,974
18	West Nusa Tenggara	134,328	164,161	214,101	256,789	313,662	361,531
19	East Nusa Tenggara	80,984	114,660	147,138	177,963	220,644	254,360
20	West Kalimantan	147,637	190,980	226,268	277,725	334,247	379,517
21	Central Kalimantan	80,222	99,635	121,140	144,825	167,201	187,099
22	South Kalimantan	150,011	186,811	224,565	265,180	305,684	343,101
23	East Kalimantan	183,540	218,933	258,704	303,522	344,498	386,903
24	North Kalimantan	40,077	46,223	53,690	60,244	66,251	74,044
25	North Sulawesi	109,696	127,309	143,976	155,365	174,275	187,540
26	Central Sulawesi	110,522	134,511	153,622	180,478	207,146	238,781
27	South Sulawesi	311,368	404,283	486,021	589,376	674,525	767,233
28	Southeast Sulawesi	85,066	108,528	134,398	165,900	198,044	230,572
29	Gorontalo	50,822	62,035	68,036	79,891	89,322	97,604
30	West Sulawesi	39,627	54,302	69,947	88,001	106,579	124,855
31	Maluku	54,481	68,997	85,466	102,228	119,809	137,921
32	North Maluku	42,875	49,239	62,267	73,121	81,782	94,949
33	West Papua	33,730	42,773	54,026	67,386	78,525	94,705
34	Papua	101,995	120,282	139,738	162,323	184,889	197,130
	Indonesia	10,028,638	12,331,842	14,557,158	17,087,133	19,551,483	21,836,747

**Table 4:** Projection of retinopathy due to diabetes in Indonesia, 2020–2045, by province.

No	Province	Number of diabetes cases projection					
		2020	2025	2030	2035	2040	2045
1	Aceh	98,700	127,750	159,529	195,052	230,667	268,241
2	North Sumatera	289,691	364,537	439,705	518,383	603,450	666,602
3	West Sumatera	103,566	132,301	163,367	200,377	235,094	270,509
4	Riau	129,844	177,243	230,245	285,648	342,541	406,309
5	Jambi	56,029	71,489	88,703	105,597	125,290	147,476
6	South Sumatera	141,942	189,494	242,465	300,038	362,633	423,436
7	Bengkulu	32,932	42,442	50,298	59,895	70,527	84,233
8	Lampung	129,996	168,569	210,127	251,001	294,855	329,169
9	Bangka Belitung	32,654	40,337	49,342	58,439	66,622	76,392
10	Riau Island	49,823	66,966	87,037	111,241	139,174	170,437
11	Jakarta	359,356	418,548	484,480	536,299	593,362	642,767
12	West Java	1,082,690	1,303,923	1,520,832	1,752,454	1,982,192	2,201,365
13	Central Java	744,503	901,062	1,043,174	1,198,128	1,326,364	1,456,802
14	Yogyakarta	120,805	146,409	174,421	204,982	242,893	281,464
15	East Java	977,496	1,156,990	1,315,677	1,464,335	1,623,196	1,775,807
16	Banten	285,147	340,084	422,326	484,547	557,553	626,210
17	Bali	109,093	139,530	167,948	201,101	228,883	266,693
18	West Nusa Tenggara	76,880	93,955	122,537	146,969	179,519	206,916
19	East Nusa Tenggara	46,350	65,624	84,212	101,854	126,282	145,579
20	West Kalimantan	84,498	109,304	129,501	158,951	191,301	217,210
21	Central Kalimantan	45,914	57,024	69,333	82,888	95,695	107,083
22	South Kalimantan	85,856	106,919	128,526	151,771	174,953	196,368
23	East Kalimantan	105,046	125,303	148,065	173,716	197,168	221,438
24	North Kalimantan	22,938	26,455	30,729	34,480	37,918	42,378
25	North Sulawesi	62,783	72,863	82,402	88,921	99,744	107,335
26	Central Sulawesi	63,255	76,985	87,923	103,294	118,557	136,663
27	South Sulawesi	178,206	231,385	278,166	337,320	386,054	439,113
28	Southeast Sulawesi	48,686	62,114	76,920	94,950	113,347	131,964
29	Gorontalo	29,087	35,505	38,939	45,725	51,122	55,862
30	West Sulawesi	22,680	31,079	40,033	50,366	60,999	71,459
31	Maluku	31,182	39,490	48,915	58,509	68,571	78,937
32	North Maluku	24,539	28,181	35,637	41,850	46,807	54,343
33	West Papua	19,305	24,481	30,921	38,567	44,942	54,203
34	Papua	58,375	68,842	79,977	92,903	105,818	112,824
	Indonesia	5,739,732	7,057,933	8,331,558	9,779,549	11,189,980	12,497,915

**Figure 2:**
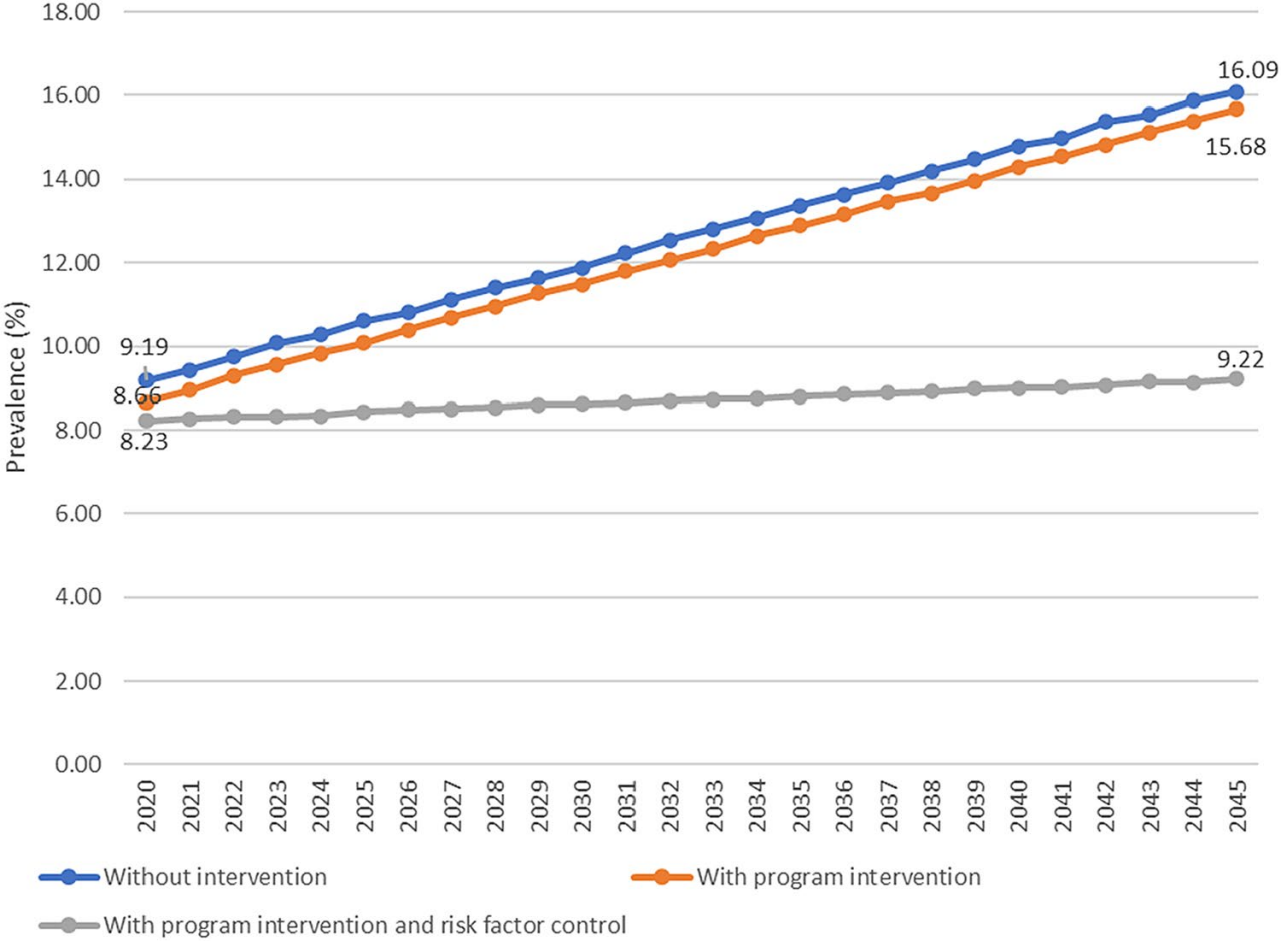
Projection of diabetes prevalence in Indonesia in three scenarios, 2020–2045.

In Fig. 2, it is known that the prevalence of Diabetes is projected at 16.09% in 2045 without intervention and will be lower to 15.68%, or reduced by 5.54%, if the intervention is carried out to increase the coverage of villages with Posbindu and SPM of Diabetes services to 100%. The prevalence will be even lower to 9.22% or reduced by 42.69% if the program intervention is added by preventing the rise of the risk factors (overweight, obesity, central obesity, and consumption of fatty foods).

**Figure 3:**
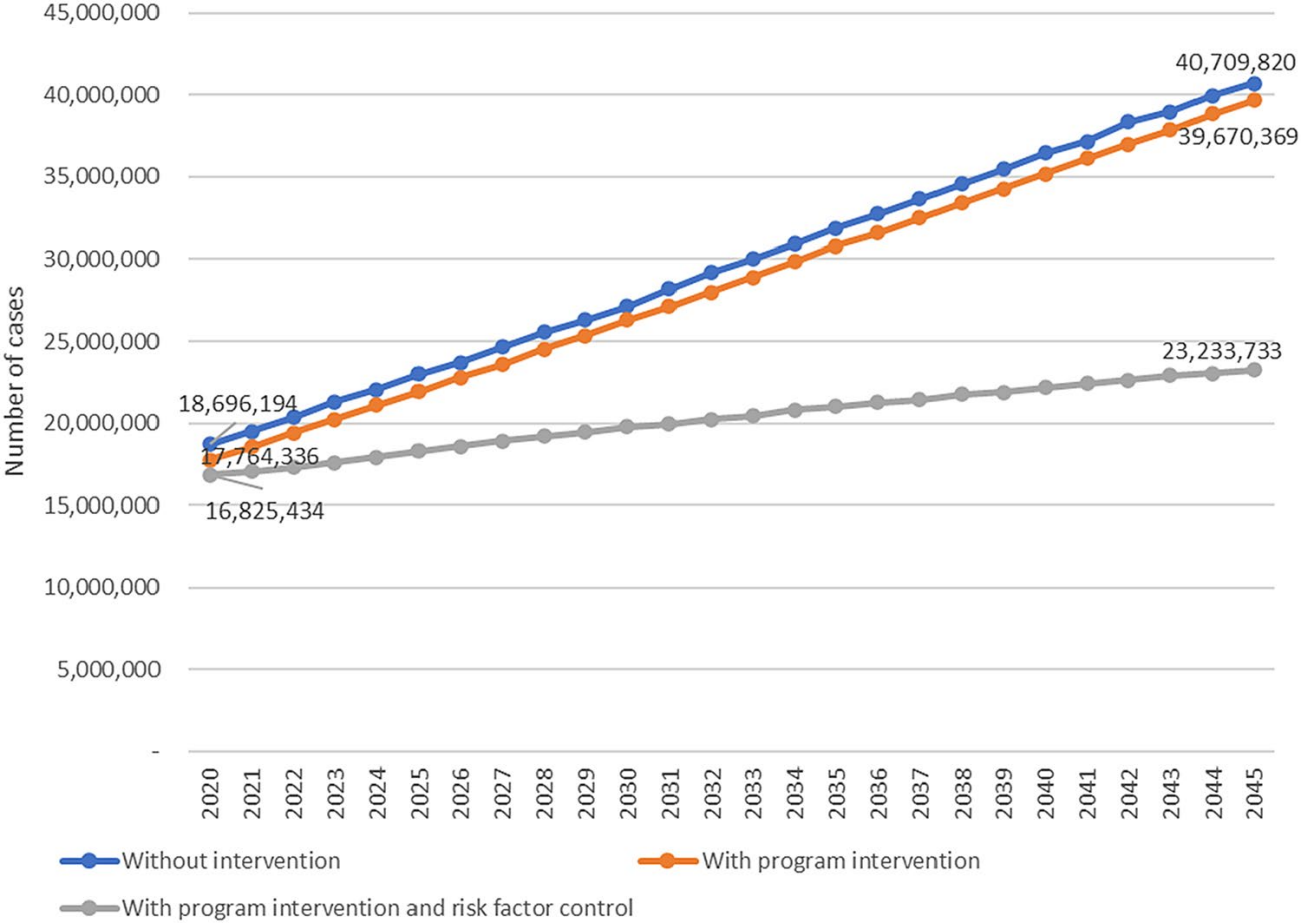
Projection of diabetes cases in Indonesia in three scenarios, 2020–2045.

The cases of Diabetes in 2045 is estimated at 40.7 million without intervention. If with the intervention of increasing the program of village with Posbindu and SPM of Diabetes services, the cases are reduced to 39.6 million cases. The cases are even lower if the program is added to halt the increase of risk factors (overweight, obesity, central obesity, consumption of fatty foods), then cases become 23.2 million (Fig. 3).

### Projection of mortality due to diabetes

**Figure 4:**
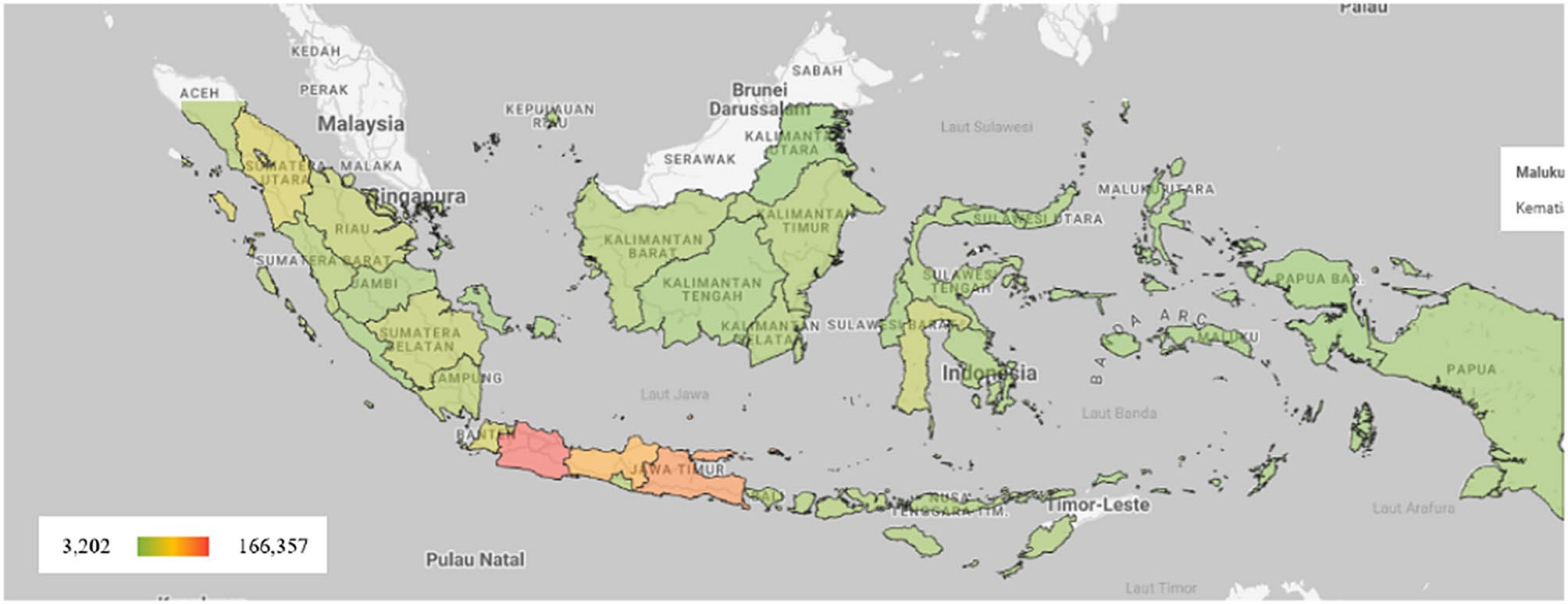
Projection of number of deaths due to diabetes in Indonesia, 2045.

The number of deaths due to Diabetes in Indonesia and each province is estimated to increase quite high in 2020–2045. Nationally, the number of deaths due to Diabetes increased from 433,752 in 2020 to 944,468 in 2045 (Fig. 4, Table 5). Stroke deaths among Diabetes cases increased from 52,397 in 2020 to 114,092 in 2045. Deaths from IHD among Diabetes cases increased from 35,351 in 2020 to 76,974 in 2045. Meanwhile, deaths from chronic kidney disease among Diabetes cases rose from 29,061 in 2020 to 63,279 in 2045. Additionally, deaths due to Diabetic Ketoacidosis (DKA) among Diabetes cases rose from 162,382 to 353,576. The number of deaths from Diabetes and its complications increased by 117% over 25 years or an average of 4.7% per year (Tables 6, 7, 8 and 9).

**Table 5:** Projection of deaths due to diabetes in Indonesia, 2020–2045.

No	Province	Projection of number of deaths due to diabetes					
		2020	2025	2030	2035	2040	2045
1	Aceh	7459	9654	12,056	14,740	17,432	20,271
2	North Sumatera	21,892	27,548	33,228	39,174	45,603	50,375
3	West Sumatera	7826	9998	12,346	15,143	17,766	20,442
4	Riau	9812	13,394	17,400	21,586	25,886	30,705
5	Jambi	4234	5402	6703	7980	9468	11,145
6	South Sumatera	10,727	14,320	18,323	22,674	27,404	31,999
7	Bengkulu	2489	3207	3801	4526	5330	6365
8	Lampung	9824	12,739	15,879	18,968	22,282	24,875
9	Bangka Belitung	2468	3048	3729	4416	5035	5773
10	Riau Island	3765	5061	6577	8406	10,517	12,880
11	Jakarta	27,157	31,630	36,612	40,528	44,840	48,574
12	West Java	81,819	98,538	114,929	132,433	149,794	166,357
13	Central Java	56,262	68,093	78,833	90,543	100,233	110,091
14	Yogyakarta	9,129	11,064	13,181	15,490	18,355	21,270
15	East Java	73,869	87,434	99,426	110,660	122,665	134,198
16	Banten	21,549	25,700	31,915	36,617	42,134	47,323
17	Bali	8244	10,544	12,692	15,197	17,297	20,154
18	West Nusa Tenggara	5810	7100	9260	11,106	13,566	15,637
19	East Nusa Tenggara	3503	4959	6364	7697	9543	11,001
20	West Kalimantan	6386	8260	9786	12,012	14,457	16,415
21	Central Kalimantan	3470	4309	5239	6264	7232	8092
22	South Kalimantan	6488	8080	9713	11,469	13,221	14,840
23	East Kalimantan	7938	9469	11,189	13,128	14,900	16,734
24	North Kalimantan	1733	1999	2322	2606	2865	3202
25	North Sulawesi	4745	5506	6227	6720	7538	8111
26	Central Sulawesi	4780	5818	6644	7806	8959	10,328
27	South Sulawesi	13,467	17,486	21,021	25,491	29,174	33,184
28	Southeast Sulawesi	3679	4694	5813	7175	8566	9973
29	Gorontalo	2198	2683	2943	3455	3863	4221
30	West Sulawesi	1714	2349	3025	3806	4610	5400
31	Maluku	2356	2984	3697	4422	5182	5965
32	North Maluku	1854	2130	2693	3163	3537	4107
33	West Papua	1459	1850	2337	2915	3396	4096
34	Papua	4411	5202	6044	7021	7997	8526
	Indonesia	433,752	533,368	629,616	739,041	845,627	944,468

**Table 6:** Projection of deaths due to stroke among *Diabetes* cases in Indonesia, 2020–2045.

No	Province	Projection of number of deaths					
		2020	2025	2030	2035	2040	2045
1	Aceh	901	1166	1456	1781	2106	2449
2	North Sumatera	2645	3328	4014	4732	5509	6085
3	West Sumatera	945	1208	1491	1829	2146	2469
4	Riau	1185	1618	2102	2608	3127	3709
5	Jambi	511	653	810	964	1144	1346
6	South Sumatera	1296	1730	2213	2739	3310	3865
7	Bengkulu	301	387	459	547	644	769
8	Lampung	1187	1539	1918	2291	2692	3005
9	Bangka Belitung	298	368	450	533	608	697
10	Riau Island	455	611	795	1016	1271	1556
11	Jakarta	3281	3821	4423	4896	5417	5868
12	West Java	9884	11,903	13,883	15,998	18,095	20,096
13	Central Java	6796	8226	9523	10,938	12,108	13,299
14	Yogyakarta	1103	1337	1592	1871	2217	2569
15	East Java	8923	10,562	12,011	13,368	14,818	16,211
16	Banten	2603	3,105	3855	4423	5090	5717
17	Bali	996	1,274	1533	1836	2089	2435
18	West Nusa Tenggara	702	858	1119	1342	1639	1889
19	East Nusa Tenggara	423	599	769	930	1153	1329
20	West Kalimantan	771	998	1182	1451	1746	1983
21	Central Kalimantan	419	521	633	757	874	978
22	South Kalimantan	784	976	1173	1385	1597	1793
23	East Kalimantan	959	1144	1352	1586	1800	2021
24	North Kalimantan	209	242	281	315	346	387
25	North Sulawesi	573	665	752	812	911	980
26	Central Sulawesi	577	703	803	943	1082	1248
27	South Sulawesi	1627	2112	2539	3079	3524	4009
28	Southeast Sulawesi	444	567	702	867	1035	1205
29	Gorontalo	266	324	355	417	467	510
30	West Sulawesi	207	284	365	460	557	652
31	Maluku	285	360	447	534	626	721
32	North Maluku	224	257	325	382	427	496
33	West Papua	176	223	282	352	410	495
34	Papua	533	628	730	848	966	1030
	Indonesia	52,397	64,431	76,058	89,276	102,152	114,092

**Table 7:** Projection of deaths due to ischemic heart disease among diabetes cases in Indonesia, 2020–2045.

No	Province	Projection of number of deaths					
		2020	2025	2030	2035	2040	2045
1	Aceh	608	787	983	1201	1421	1652
2	North Sumatera	1784	2245	2708	3193	3717	4106
3	West Sumatera	638	815	1006	1234	1448	1666
4	Riau	800	1,092	1418	1759	2110	2502
5	Jambi	345	440	546	650	772	908
6	South Sumatera	874	1,167	1493	1848	2233	2608
7	Bengkulu	203	261	310	369	434	519
8	Lampung	801	1,038	1294	1546	1816	2027
9	Bangka Belitung Island	201	248	304	360	410	470
10	Riau Island	307	412	536	685	857	1050
11	Jakarta	2213	2578	2984	3303	3654	3959
12	West Java	6668	8031	9367	10,793	12,208	13,558
13	Central Java	4585	5550	6425	7379	8169	8972
14	Yogyakarta	744	902	1074	1262	1496	1734
15	East Java	6020	7126	8103	9019	9997	10,937
16	Banten	1756	2095	2601	2984	3434	3857
17	Bali	672	859	1034	1239	1410	1643
18	West Nusa Tenggara	474	579	755	905	1106	1274
19	East Nusa Tenggara	285	404	519	627	778	897
20	West Kalimantan	520	673	798	979	1178	1338
21	Central Kalimantan	283	351	427	511	589	660
22	South Kalimantan	529	659	792	935	1078	1209
23	East Kalimantan	647	772	912	1070	1214	1364
24	North Kalimantan	141	163	189	212	234	261
25	North Sulawesi	387	449	508	548	614	661
26	Central Sulawesi	390	474	542	636	730	842
27	South Sulawesi	1098	1425	1713	2078	2378	2704
28	Southeast Sulawesi	300	383	474	585	698	813
29	Gorontalo	179	219	240	282	315	344
30	West Sulawesi	140	191	247	310	376	440
31	Maluku	192	243	301	360	422	486
32	North Maluku	151	174	219	258	288	335
33	West Papua	119	151	190	238	277	334
34	Papua	360	424	493	572	652	695
	Indonesia	35,351	43,470	51,314	60,232	68,919	76,974

**Table 8:** Projection of deaths due to chronic kidney disease among diabetes cases in Indonesia, 2020–2045.

No	Province	Projection of number of deaths					
		2020	2025	2030	2035	2040	2045
1	Aceh	500	647	808	988	1168	1358
2	North Sumatera	1467	1846	2226	2625	3055	3375
3	West Sumatera	524	670	827	1015	1190	1370
4	Riau	657	897	1166	1446	1734	2057
5	Jambi	284	362	449	535	634	747
6	South Sumatera	719	959	1228	1519	1836	2144
7	Bengkulu	167	215	255	303	357	426
8	Lampung	658	853	1064	1271	1493	1667
9	Bangka Belitung Island	165	204	250	296	337	387
10	Riau Island	252	339	441	563	705	863
11	Jakarta	1819	2119	2453	2715	3004	3254
12	West Java	5482	6602	7700	8873	10,036	11,146
13	Central Java	3770	4562	5282	6066	6716	7376
14	Yogyakarta	612	741	883	1038	1230	1425
15	East Java	4949	5858	6662	7414	8219	8991
16	Banten	1444	1722	2138	2453	2823	3171
17	Bali	552	706	850	1018	1159	1350
18	West Nusa Tenggara	389	476	620	744	909	1048
19	East Nusa Tenggara	235	332	426	516	639	737
20	West Kalimantan	428	553	656	805	969	1100
21	Central Kalimantan	232	289	351	420	485	542
22	South Kalimantan	435	541	651	768	886	994
23	East Kalimantan	532	634	750	880	998	1121
24	North Kalimantan	116	134	156	175	192	215
25	North Sulawesi	318	369	417	450	505	543
26	Central Sulawesi	320	390	445	523	600	692
27	South Sulawesi	902	1172	1408	1708	1955	2223
28	Southeast Sulawesi	247	314	389	481	574	668
29	Gorontalo	147	180	197	232	259	283
30	West Sulawesi	115	157	203	255	309	362
31	Maluku	158	200	248	296	347	400
32	North Maluku	124	143	180	212	237	275
33	West Papua	98	124	157	195	228	274
34	Papua	296	349	405	470	536	571
	Indonesia	29,061	35,736	42,184	49,516	56,657	63,279

**Table 9:** Projection of deaths due to diabetic ketoacidosis among diabetes cases in Indonesia, 2020–2045.

No	Province	Projection of number of deaths					
		2020	2025	2030	2035	2040	2045
1	Aceh	2792	3614	4513	5518	6526	7589
2	North Sumatera	8196	10,313	12,440	14,665	17,072	18,859
3	West Sumatera	2930	3743	4622	5669	6651	7653
4	Riau	3673	5014	6514	8081	9691	11,495
5	Jambi	1585	2022	2509	2987	3545	4172
6	South Sumatera	4016	5361	6860	8488	10,259	11,979
7	Bengkulu	932	1201	1423	1694	1995	2383
8	Lampung	3678	4769	5945	7101	8342	9312
9	Bangka Belitung Island	924	1141	1396	1653	1885	2161
10	Riau Island	1410	1895	2462	3147	3937	4822
11	Jakarta	10,166	11,841	13,706	15,172	16,787	18,184
12	West Java	30,630	36,889	43,026	49,578	56,078	62,278
13	Central Java	21,063	25,492	29,512	33,896	37,524	41,214
14	Yogyakarta	3418	4142	4935	5799	6872	7963
15	East Java	27,654	32,732	37,222	41,427	45,922	50,239
16	Banten	8067	9621	11,948	13,708	15,774	17,716
17	Bali	3086	3947	4751	5689	6475	7545
18	West Nusa Tenggara	2175	2658	3467	4158	5079	5854
19	East Nusa Tenggara	1311	1857	2382	2882	3573	4119
20	West Kalimantan	2391	3092	3664	4497	5412	6145
21	Central Kalimantan	1299	1613	1961	2345	2707	3029
22	South Kalimantan	2429	3025	3636	4294	4950	5555
23	East Kalimantan	2972	3545	4,189	4915	5578	6265
24	North Kalimantan	649	748	869	975	1073	1199
25	North Sulawesi	1776	2061	2331	2516	2822	3037
26	Central Sulawesi	1790	2178	2487	2922	3354	3866
27	South Sulawesi	5042	6546	7870	9543	10,922	12,423
28	Southeast Sulawesi	1377	1757	2176	2686	3207	3733
29	Gorontalo	823	1004	1102	1294	1446	1580
30	West Sulawesi	642	879	1133	1425	1726	2022
31	Maluku	882	1117	1384	1655	1940	2233
32	North Maluku	694	797	1008	1184	1324	1537
33	West Papua	546	693	875	1091	1271	1533
34	Papua	1651	1948	2263	2628	2994	3192
	Indonesia	162,382	199,675	235,707	276,671	316,574	353,576

At the provincial level, deaths due to Diabetes and its three complications in 2045 are highest in West Java province with 166,357 deaths due to Diabetes, 3,202 deaths from stroke among Diabetes, 13,558 deaths from IHD among Diabetes, and 11,146 deaths from CKD among Diabetes. The lowest mortality was in North Kalimantan province with 3,202 deaths due to Diabetes, 387 deaths from stroke among Diabetes, 261 deaths from IHD among Diabetes, and 215 deaths from CKD among Diabetes Tables 6, 7, 8 and 9).

**Figure 5:**
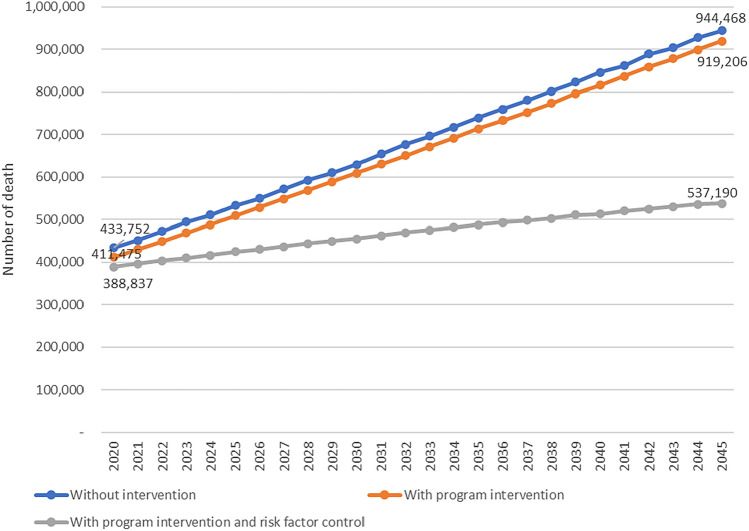
Projection of deaths due to diabetes in three scenarios in Indonesia, 2020–2045.

Figure 5 shows that the number of deaths due to Diabetes in 2045 is estimated at 944,468 if without intervention and lower to 919,206 (reduced by 2.67%) if program improvement interventions are carried out and to 537,190 (reduced by 43.12%) if program improvement is added with controlling of the risk factors increase.

## Discussion

The result of the study shows that the prevalence of Diabetes in Indonesia increased from 9.19% in 2020 (18.69 million cases) to 16.09% in 2045 (40.7 million cases) or an increase of 75.1% over 25 years, or an average of 3% per year. The province with the highest prevalence in 2045 is Jakarta (23.11%) and the lowest is East Nusa Tenggara province (8.91%). The largest number of cases in 2045 is in West Java Province (7,170,569 cases) and the lowest is in North Kalimantan Province (138,038 cases). The results of this study indicate a large increase in the prevalence and number of Diabetes cases in Indonesia, if adequate prevention and control of the NCDs risk factors programs are not carried out. Jakarta Province is an urban area which have higher Diabetes risk factors so that the prevalence is the highest. NTT Province is a rural province with a lower risk of Diabetes, so the prevalence is the lowest. The size of cases corresponds to the magnitude of the prevalence and the number of adult populations. The number of Diabetes cases is according to the prevalence and number of people aged 15 years and over, so provinces with large populations tend to have a larger number of Diabetes cases. West Java Province is the largest province in Indonesia so that the number of cases is the largest, while North Kalimantan province is the province with the smallest population so that the number of Diabetes cases is also the lowest.

The increase in the prevalence of Diabetes in Indonesia is almost the same as the results of other studies. In Indonesia, Nuryati’s research in 2012^27^ shows that the prevalence diabetes among adults in Indonesia 8.04%. This study was a cross sectional study using secondary data from Basic Health Research 2007 with respondents above 18 years using oral glucose tolerance test. Nuryati’s study used the same as this study from Basic Health Research but with different period (2007 and 2018 data) so the prevalence was quite same. But, the projection from this study is higher that projection of Nurhayati^16^ that by 2020 the prevalence was 8.71% in Indonesia and 9.49% in 2024. Nurhayati’s study used a literature review based on Institute of Health Metric and Evaluation (IHME) data which was based on relative risk modelling using regression analysis. This projection is different from this study which used not only risk factors but also Diabetes programs. It indicates that the programs influence the burden of diabetes in Indonesia.

In Thailand, Mahikul et al.^28^ reported that Diabetes prevalence is predicted to increase from 6.5% in 2015 to 10.69% in 2035 or an increase of 64.4% over 20 years or 3.2% per year. According to data from the Institute of Health Metric and Evaluation, the prevalence of Diabetes in Indonesia in 2019 is estimated at 3.98% of the entire population or 10.33 million cases^1^. In China, research of Pan et al.^29^ in a systematic review 1987–2007 reported that the prevalence of Diabetes in China in 2009 was 3.9% (urban 5.2%, rural 2.9%) and is predicted to increase to 5.4% (urban 6.9%, rural 3.8%) in 2016, or an increase of 38.4% over 7 years or an annual increase of 4.6%. Meanwhile, the number of Diabetes cases is projected to increase from 53.1 million cases in 2009 to 76.1 million cases in 2016. In Sweden, Andersson et al.^30^ reported that the prevalence of Diabetes increased from 5.8% in 2007 to 6.8% in 2013 (2013) and will rise to 10.4% in 2050 or an increase of 79.3% over 1.8% per year. The number of cases is predicted to increase to 940,000 and every 1% increase in annual incidence will result in an increase of 12.6% prevalence and 1,136 000 cases.

In the United States by Boyle et al.^19^ where if Diabetes mortality is high, then Diabetes prevalence increases from 14% in 2010 to 21% in 2050 (increase of 50% or 1.25% per year) and to 33% in 2050 (increase of 135%) or 3.3% per year) for 40 years if mortality is low. Rowley et al.^18^ in the US The prevalence of Diabetes will increase by 54% to more than 54.9 million US population between 2015 and 2030. Diabetes—related annual deaths will rise by 38% to 385,800. Another study by Mainous et al.^31^ in the United States, projections of Diabetes burden based on individual risk prevalence show that the total burden of Diabetes is estimated at 11.5% (25.4 million) in 2011, 13.5% (32.6 million) in 2021, and 14.5% (37.7 million) in 2031 or an increase of 26% over 20 years with an average increase of 1.3% per year. Wild et al.^32^ projected that diabetes prevalence is estimated at 2.8% in 2000 and increases to 4.4% by 2030 worldwide, or an increase of 57.1% from the prevalence over 30 years, with an average of 1.9% increase per year. The number of people with diabetes in the world is expected to increase from 171 million cases in 2000 to 366 million cases in 2030.

Based on the scenarios, the results of this study show that the prevalence of Diabetes by 2045 was 16.09% and can be reduced to 15.68%, or reduced by 5.54%, if program intervention namely increase of the coverage of villages with Posbindu and SPM of Diabetes services to 100%. This figure can be lowered again to 9.22% or reduced by 42.69% if the program intervention is added with prevention of risk factors (*overweight*, obesity, central obesity and consumption of fatty foods). These results show that existing program interventions (Posbindu village and SPM of Diabetes services play a role in reducing the prevalence of Diabetes but not so large. The reduction will be much greater if prevention of the main risk factors for Diabetes are overweight, obesity, central obesity, and consumption of fatty foods.

To reduce Diabetes cases, efforts are needed to control risk factors that positively affect Diabetes projections, namely overweight, obesity, central obesity, and consumption of fatty foods. These control efforts are carried out through increasing education, physical activity, and efforts to change the pattern of consumption of fatty foods into healthy foods (enough fruits and vegetables). Efforts to halt the prevalence rate of these four risk factors can be made through a combination of physical activity and a healthy diet. Intervention targets need to be more specific to at-risk populations. Research by Gregg et al.^33^ in the United States, shows that by 2030 it is projected that 4.6 million incidences and 3.6 million cases of Diabetes prevalence or reducing the prevalence rate by 14% can be prevented by a combination of prevention strategies. This prevention strategy is developed with structured lifestyle interventions for high-risk (pre-diabetic), moderate-risk, and general populations.

The Ministry of Health of Indonesia needs to make policies and programs to prevent risk factors. The program can be through educational efforts through the Healthy Living Community Movement (GERMAS) and healthy behavior in the community. It is necessary to increase 100% Village with Posbindu. In addition, the achievement of Diabetes health service SPM becomes 100% every year. National Planning Bureau needs to include efforts to control Diabetes risk factors, especially *overweight*, obesity, central obesity, and unhealthy consumption patterns in health program plans in Indonesia and provide sufficient budget related to Posbindu and Diabetes health service SPM.

The results showed that the projected number of deaths due to Diabetes in Indonesia increased from 433,752 deaths in 2020 to 944,468 in 2045. Stroke deaths in Diabetes increased from 52,397 in 2020 to 114,092 in 2045. Deaths from IHD in Diabetes increased from 35,351 in 2020 to 76,974 in 2045. Meanwhile, deaths from chronic kidney disease in Diabetes increased from 29,061 in 2020 to 63,279 in 2045. The number of deaths from Diabetes and its complications increased by 117% over 25 years or an average of 4.7% per year.

These results indicate that Diabetes is one of the highest causes of death in Indonesia. Based on data from the Institute of Health Metric and Evaluation, deaths from Diabetes in Indonesia in 2019 amounted to 40.98 per 100,000 population or 106,333 deaths. It has the largest increase of all other causes of death for 128.7% from 1990^1^. Meanwhile, based on data from the 2015 Sample Registration System, Diabetes is the third highest cause of death in Indonesia after stroke and ischemic heart disease with a proportion of 7.8%^34^, an increase from 5.7% in 2007^2^.

In Singapore, research by Tan et al.^17^ shows that Diabetes complications in 2050 in the form of acute myocardial infarction will increase from 9300 deaths (2019) to 16,400 (2050), the number of stroke increase from 7300 to 12,800, the number of end-stage kidney disease from 1700 to 2700. This number increased by an average of 76.3% over 30 years. In Thailand, Mahikul et al.^28^ in their study predicted death in undiagnosed Diabetes 10 times greater than undiagnosed Diabetes. The positive screening rate decreased mortality in women aged 15–34 years at 10 years. This indicates the importance of blood sugar screening so that people can be aware of the dangers of diabetes and can make prevention and control efforts independently. Research by Foreman et al.^35^ shows that deaths from Diabetes in the world amounted to 1,437,000 in 2016 to 2,971,000 in 2040, or an increase of 106.7% over 24 years, with an average increase of 4.4% per year. Deaths from Diabetes -related kidney disease in the world 500,000 in 2016 to 1380 in 2040. Stroke deaths in the world were 5,528,000 in 2016 to 5973 in 2040. The number of ischemic heart disease deaths worldwide was 9,480,000 in 2016 to 10,872,000 in 2040.

Deaths from Diabetes and its complications need to be suppressed with appropriate primary, secondary, and tertiary prevention. Ministry of Health to improve such prevention adequately. On primary prevention to prevent complications in people with diabetes through diet modification and a healthy lifestyle. In secondary prevention, treatment of Diabetes and its complications needs to be provided to all patients using the latest technology. Increased achievement of SPM of Diabetes Health services. In tertiary prevention, rehabilitation for advanced cases such as diabetic foot care needs to be expanded, including home care services.

To reduce the fatality of diabetes due to its complications such as Stroke, Ischemic heart disease, Chronic kidney disease and immediate fatality due to Diabetic Ketoacidosis, interventions of the disease should be enhanced across the regions in Indonesia. Chronic disease management program (Prolanis), number of primary health care facilities providing optimal diabetes services (NCD integrated services), and the participation of healthy life movement (GERMAS) should be strengthened. Meanwhile, achievement of blood glucose targets under Prolanis and the percentage of diabetic patients receiving scheduled screenings and counselling with specialists should be increased.

This study has several limitations in terms of quality and representation of research data. This is due to inconsistent data, missing data, and program coverage data exceeding 100%. For inconsistent data, projections use their mean and standard deviation. For data that exceeds the target of 100%, the data is fulfilled to a maximum of 100%. There are 3.3–34.6% missing data that can reduce data quality in making projections. For these circumstances, the data is filled in using the average province of the district so that the data does not deviate from the actual condition.

Data representation for village with Posbindu, Pandu, and SPM of Diabetes services, and SPM of screening are routine data that is inputted by district government which tends to overestimate, because there is no individual data. However, this data is an official release from the Ministry of Health so it can still be used. The data analyzed in this study is aggregate data at the district level (205 districts/cities out of 514 districts/cities) to estimate the burden of Diabetes at the district, provincial, and national levels. This can cause actual projections to vary more than the results of this study because not all district data are analyzed in the preparation of the model. However, with a provincial MAPE value of 13% that is good at making projections at the provincial level and MAPE at the district / city level, the projection is still quite feasible to estimate conditions in the district/city.

## Conclusion

Diabetes morbidity and mortality in Indonesia is projected to rise significantly in Indonesia from 2020 to 2045. The prevalence increases 75.1% over 25 years, with an average of 3% from prevalence per year. The number of deaths from Diabetes and its complications increased by 117% over 25 years or an average of 4.7% per year. Morbidity and mortality can be reduced by intervention of several programs (Village with NCD Post/Posbindu, standard service of diabetes) and risk factors control (overweight, obesity, central obesity, and fatty food consumption). It is recommended to Ministry of Health and health policy makers to use this study result as source of planning and evaluation of diabetes prevention and control program. I need to strengthen the program of risk factor monitoring trough Posbindu, achieve target of minimum standard services of diabetes, and increase healthy lifestyle including physical activity and healthy diet to control overweight and obesity.

## Data Availability

Data of the research is available and can be shared on request to Anhari Achadi at aachadi@gmail.com.

## Abbreviations

DM: Diabetes Mellitus
MAPE: Absolute Mean Percentage Error
NCD: Non Communicable Disease
Pandu: *Pelayanan Terpadu* (NCD integrated services at Primary Health Center)
Posbindu: *Pos Pembinaan Terpadu* (NCD integrated post at community)
Prolanis: *Program pelayanan penyakit kronis* (Chronic disease management)
SPM: *Standar Pelayanan Minimal* (Minimum Standard Services)

## References

[1] IHME. *Burden of Disease, 2019*. Available at https://vizhub.healthdata.org/gbd-compare/ (2020).

[2] Kemenkes RI. Report on result of national basic health research (Riskesdas) 2007. Available at https://repository.badankebijakan.kemkes.go.id/id/eprint/4386/1/Report%20on%20Result%20of%20National%20Basic%20Health%20Research%202007.pdf (2008).

[3] Kemenkes RI. *Laporan Nasional Riset Kesehatan Dasar (Riskesdas) 2013*. Available at https://repository.badankebijakan.kemkes.go.id/id/eprint/4467/1/Laporan_riskesdas_2013_final.pdf (2013).

[4] Kemenkes RI. *Laporan Nasional Riset Kesehatan Dasar (Riskesdas) 2018*. Available at https://repository.badankebijakan.kemkes.go.id/id/eprint/3514/1/Laporan%20Riskesdas%202018%20Nasional.pdf (2019).

[5] WHO (2001). Summary Surveillance of Risk Factors for Noncommunicable Diseases The WHO STEPwise Approach.

[6] Perkeni. *Pedoman pengelolaan dan pencegahan diabetes melitus tipe 2 dewasa di Indonesia 2019*. Available at https://pbperkeni.or.id/wp-content/uploads/2021/06/Pedoman-Pengelolaan-DM-Tipe-2-Dewasa-di-Indonesia-eBook-PDF.pdf (PB Perkeni, Jakarta, 2019).

[7] Kemenkes, R. I. *Pedoman Umum Pencegahan dan Pengendalian DM Tipe 2* (Jakarta, 2016).

[8] Peters R, Ee N, Peters J, Beckett N, Booth A, Rockwood K (2018). Common risk factors for major noncommunicable disease, a systematic overview of reviews and commentary: The implied potential for targeted risk reduction. Ther. Adv. Vaccines.

[9] Zhang H, Ni J, Yu C, Wu Y, Li J, Liu J (2019). Sex-based differences in diabetes prevalence and risk factors: A population-based cross-sectional study among low-income adults in China. Front. Endocrinol. (Lausanne).

[10] Presiden RI (2020). Peraturan Presiden RI Nomor 18 Tahun 2020 Tentang Rencana Pembangunan Jangka Menengah Nasional Tahun 2020 - 2024.

[11] Kemenkes RI (2020). Peraturan Menteri Kesehatan Republik Indonesia Nomor 21 Tahun 2020 Tentang Rencana Strategis Kementerian Kesehatan Tahun 2020-2024.

[12] Kemenkes RI (2019). Peraturan Menteri Kesehatan RI Nomor 4 Tahun 2019 tentang Standar Teknis Pemenurhan Mutu Pelayanan Dasar Pada Standar Pelayanan Minimal Bidang Kesehatan.

[13] Meng XH, Huang YX, Rao DP, Zhang Q, Liu Q (2013). Comparison of three data mining models for predicting diabetes or prediabetes by risk factors. Kaohsiung J. Med. Sci..

[14] Nai-Arun N, Moungmai R (2015). Comparison of classifiers for the risk of diabetes prediction. Procedia Comput. Sci..

[15] Tjokroprawiro A (1993). Diabetes Mellitus di dalam-Masyarakat Indonesia. Bul. Penelit. Kesehat..

[16] Nurhayati, H.-W. *Projected number of people with diabetes Indonesia 2017–2024.* Available at https://www.statista.com/statistics/1052625/indonesia-diabetes-projection/ (Statista, 2020).

[17] Tan KW, Dickens BSL, Cook AR (2020). Projected burden of type 2 diabetes mellitus-related complications in Singapore until 2050: A Bayesian evidence synthesis. BMJ Open Diabetes Res. Care.

[18] Rowley WR, Bezold C, Arikan Y, Byrne E, Krohe S (2017). Diabetes 2030: Insights from yesterday, today, and future trends. Popul. Health Manag..

[19] Boyle JP, Thompson TJ, Gregg EW, Barker LE, Williamson DF (2010). Projection of the year 2050 burden of diabetes in the US adult population: Dynamic modeling of incidence, mortality, and prediabetes prevalence. Popul. Health Metr..

[20] Hastono, S. P. *Analisis Data. Depok: Fakultas Kesehatan Masyarakat Universitas Indonesia* (Depok, 2006).

[21] Kleinbaum DG (1998). Applied Regression Analysis and Other Multivariable Methods.

[22] Purwata TE (2011). High TNF-alpha plasma levels and macrophages iNOS and TNF-alpha expression as risk factors for painful diabetic neuropathy. J. Pain Res..

[23] Halim A, Syumarti S, Rini M, Ratnaningsih N, Iskandar E, Sovani I (2022). Prevalence and associated factors of diabetic retinopathy in people with type 2 diabetes attending community based diabetic retinopathy screening in greater Bandung Indonesia. Int. J. Retin..

[24] Dewata DGUB, Novida H, Aryati A (2020). Profile of diabetic ketoacidosis patients at regional public hospital Dr. Soetomo in 2017. J. Berk Epidemiol..

[25] Siregar NN, Soewondo P, Subekti I, Muhadi M (2018). Seventy-two hour mortality prediction model in patients with diabetic ketoacidosis: A retrospective cohort study. J. ASEAN Fed. Endocr. Soc..

[26] Sterman JD (2004). Business Dinamics: Systems Thinking and Modeling for a Complex World.

[27] Nuryati E (2012). Faktor prediksi diabetes melitus tidak terdiagnosis ada usia dewasa di Indonesia Tahun 2011. J. Ilm Kesehat..

[28] Mahikul W, White LJ, Poovorawan K, Soonthornworasiri N, Sukontamarn P, Chanthavilay P (2019). A population dynamic model to assess the diabetes screening and reporting programs and project the burden of undiagnosed diabetes in Thailand. Int. J. Environ. Res. Public Health.

[29] Pan C, Shang S, Kirch W, Thoenes M (2010). Burden of diabetes in the adult Chinese population: A systematic literature review and future projections. Int. J. Gen. Med..

[30] Andersson T, Ahlbom A, Carlsson S (2015). Diabetes prevalence in Sweden at present and projections for year 2050. PLoS One.

[31] Mainous AG, Baker R, Koopman RJ, Saxena S, Diaz VA, Everett CJ (2007). Impact of the population at risk of diabetes on projections of diabetes burden in the United States: An epidemic on the way. Diabetologia.

[32] Wild SH, Roglic G, Green A, Sicree R, King H (2004). Global prevalence of diabetes: Estimates for the year 2000 and projections for 2030. Diabetes Care.

[33] Gregg EW, Boyle JP, Thompson TJ, Barker LE, Albright AL, Williamson DF (2013). Modeling the impact of prevention policies on future diabetes prevalence in the United States: 2010–2030. Popul. Health Metr..

[34] Kemenkes RI (2015). Indonesia: Sample registration system 2014.

[35] Foreman KJ, Marquez N, Dolgert A, Fukutaki K, Fullman N, McGaughey M (2018). Forecasting life expectancy, years of life lost, and all-cause and cause-specific mortality for 250 causes of death: Reference and alternative scenarios for 2016–40 for 195 countries and territories. Lancet.

